# Mechanism of Action of Melatonin as a Potential Adjuvant Therapy in Inflammatory Bowel Disease and Colorectal Cancer

**DOI:** 10.3390/nu16081236

**Published:** 2024-04-21

**Authors:** Abdo Jurjus, Jad El Masri, Maya Ghazi, Lemir Majed El Ayoubi, Lara Soueid, Alice Gerges Geagea, Rosalyn Jurjus

**Affiliations:** 1 Department of Anatomy, Cell Biology and Physiological Sciences, Faculty of Medicine, American University of Beirut, Beirut 1107, Lebanon; jse20@mail.aub.edu (J.E.M.); mayanghazi99@gmail.com (M.G.); lxs03@mail.aub.edu (L.S.); alicegerges@gmail.com (A.G.G.); rosalynej@gmail.com (R.J.); 2 Faculty of Medical Sciences, Lebanese University, Beirut 6573, Lebanon; majedelayoubi@gmail.com

**Keywords:** melatonin, inflammatory bowel disease, colorectal cancer, inflammation, immune system, oxidative stress

## Abstract

Inflammatory bowel disease (IBD), a continuum of chronic inflammatory diseases, is tightly associated with immune system dysregulation and dysbiosis, leading to inflammation in the gastrointestinal tract (GIT) and multiple extraintestinal manifestations. The pathogenesis of IBD is not completely elucidated. However, it is associated with an increased risk of colorectal cancer (CRC), which is one of the most common gastrointestinal malignancies. In both IBD and CRC, a complex interplay occurs between the immune system and gut microbiota (GM), leading to the alteration in GM composition. Melatonin, a neuroendocrine hormone, was found to be involved with this interplay, especially since it is present in high amounts in the gut, leading to some protective effects. Actually, melatonin enhances the integrity of the intestinal mucosal barrier, regulates the immune response, alleviates inflammation, and attenuates oxidative stress. Thereby, the authors summarize the multifactorial interaction of melatonin with IBD and with CRC, focusing on new findings related to the mechanisms of action of this hormone, in addition to its documented positive outcomes on the treatment of these two pathologies and possible future perspectives to use melatonin as an adjuvant therapy.

## 1. Introduction

Melatonin is a well-characterized neurohormone secreted in humans primarily by the pineal gland in response to the exposure of the suprachiasmatic nucleus to light and dark cycles [1]. It is also secreted by other organs, including the skin, the retina, the bone marrow, and the gastrointestinal tract (GIT). In each location, it is stimulated by specific triggers [2]. The main role of this neurohormone is to regulate the circadian rhythm, allowing the body to adapt its functions in accordance to the differences between the day and the night, like regulating core body temperature and sleep/wake cycles [3]. Moreover, melatonin is considered an important antioxidant agent. It achieves this role by directly detoxifying the accumulated reactive oxygen and/or nitrogen species, or by regulating the balance between anti- and pro-oxidant enzymes [4]. This feature has opened new insights to explore the use of this hormone in inflammatory diseases, including Inflammatory Bowel Disease (IBD) and cancers [5].

Inflammatory bowel disease (IBD), which predominately consists of Ulcerative Colitis (UC) and Crohn’s disease (CD), manifests in most patients, with diarrhea, weight loss, and abdominal pain, and it is sometimes associated with symptoms outside the GIT [6]. The pathogenesis of IBD is still not completely clear, but it is tightly associated to the immune system dysregulation caused by dysbiosis in the normal gut flora, which in turn causes GIT inflammation [7]. One of the seriously considered etiological factors of IBD is oxidative stress (OS) [8]. Hence, the antioxidant activities of melatonin have made it a noteworthy agent to be further studied as part of the treatment for IBD. Although most of the currently available studies are experimental, a few clinical applications of this hormone are reported [9,10]. Additionally, the anti-inflammatory properties of melatonin make it a potential therapeutic option that has been shown to improve digestive disorders in various GIT diseases, including IBD [11].

Furthermore, up to 15% of IBDs can lead to colorectal cancer (CRC) [12], which is the third-most common cancer and ranks second in mortality caused by cancer worldwide [13]. This trend is expected to increase in view of the increasing number of people shifting towards Western diets and lifestyles. Early detection of precancerous lesions and CRC onset, especially by colonoscopy, is of paramount importance [14]. The current therapeutic approach to CRC is based on monotherapy or a combination of chemotherapy, immunotherapy, radiation therapy, and surgery [15]. Nevertheless, due to the magnitude of the adverse effects caused by the existing therapies, new agents are being studied in order to improve clinical outcomes. In this regard, melatonin was found to have an important anti-tumoral activity by halting cancerous proliferation and fighting off metastases, especially in CRC [16]. It has gained this role through various mechanistic pathways that were experimentally studied, including the direct effect of the hormone itself on cancerous cells, or indirectly by sensitizing tumoral cells to current therapies [16,17].

Furthermore, dysbiosis is associated with most gastrointestinal diseases, including IBD and CRC [18]. Patients with IBD and CRC have a lower gut bacterial diversity compared to healthy patients. Several harmful bacteria were found to be responsible for IBD pathogenesis, such as *Firmicutes* (abundant in bare sand), *Proteobacteria*, and *Roseburia* spp., including *Roseburia faecis* and *Roseburia intestinalis* [19]. The relative increase of these species in the gut, leads to their attachment to colonic mucosa, thus affecting and increasing its permeability [20]. As for CRC, the exact pathogenesis is unclear and needs further investigation [21]. The alteration in GM is considered one of the main factors associated with the transformation of a present adenoma to carcinoma [22]. For instance, carcinogenesis can be stimulated by some bacteria, namely *Fusobacterium nucleatum*, *Firmicutes*, *Streptococcus gallolytics*, and *Bacteroidetes* [23]. In brief, the above-mentioned alteration in gut microbiome can trigger inflammatory and neoplastic processes. However, these mechanisms are not yet fully elucidated, in particular, the potential role of melatonin.

After searching PubMed, Scopus, and Web of Science for recent studies related to the effects of melatonin in IBD and in CRC, this narrative review offers an update regarding the multifactorial interaction between melatonin and these gastrointestinal diseases. The main focus of this review is on new findings related to the mechanisms of action of this hormone, in addition to its described positive outcomes on the treatment of these two pathologies.

## 2. Melatonin: Structure and Function

**Figure 1:**
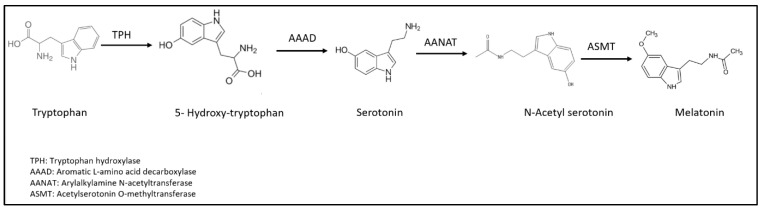
Biosynthetic pathway of melatonin through different enzymes (adopted from Tordjman et al., 2017) [25].

Melatonin, also known as 5 methoxy-N-acetlytryptamine, is a hormone secreted by mainly the pineal gland [24], most commonly known for a key role in the regulation of sleep [25]. It is derived from tryptophan, a lipophilic amino acid, that is transformed into melatonin under the activity of multiple enzymes [26]. Figure 1 presents a summarized synthetic pathway of melatonin [25].

There are numerous factors that could affect melatonin levels, such as the dark-light cycle, seasons, age, and many more [27]. Melatonin majorly regulates the circadian rhythms by acting on multiple receptors, which permits the adaptation to the environment by inducing the temporal organization of functions and linking them to the changes in the environment [25]. In general, injections of melatonin can generate fatigue and sleep quiescence, defined by a period where cells exit their normal cycle and are starved for nutrients [28,29].

The initial step of melatonin synthesis is the uptake of systemic tryptophan (Trp). A diet rich in Trp increases the levels of melatonin at different rates [30]. For instance, a study done on humans showed that samples with oral Trp had higher levels of melatonin in the blood compared to samples receiving Trp through the intraperitoneal route [31]. Moreover, a study in mice showed that the intake of a certain amount of Trp leads to a significant increase in melatonin levels, but to a limited extent, if the quantity of dietary Trp is doubled, the systemic levels of melatonin remain the same [31]. Furthermore, the intake of Trp during nighttime showed a significant decrease in the amount of melatonin. Such results highlight the importance of Trp for the synthesis of melatonin and, consequently, its effect on sleep regulation [32]. Thus, more studies are needed to elucidate the appropriate influence of Trp doses and administration on melatonin synthesis and effect.

Additionally, melatonin plays an important role in fetal development. Such results are explained by the direct effect of melatonin on the placenta resulting in the programming of the biological clock of the fetus [33]. As shown in Figure 1, Arylalkylamine N-acetyltransferase (AANAT) and Acetylserosine O-methyltransferase (ASMT) are responsible for the conversion of serotonin to melatonin [34]. These enzymes are present in the human placenta in the first trimester. Moreover, melatonin increases significantly the secretion of human chorionic gonadotropin (Hcg) produced by trophoblast tissue typically present in embryos [35]. Such findings highlight the potential effect of melatonin by acting on different organs and exerting multiple functions in the human body.

## 3. Melatonin in the Gut

Melatonin was reported to be present in different locations such as the retina, skin, and bone marrow, with high levels found in the gut [25]. More precisely, experiments showed that melatonin concentration in the gut was 400 times higher than in the pineal gland [36]. These high levels can be explained by the presence of many gut-related factors responsible for melatonin production [37]. The high concentrations of melatonin are explained by its production by many gut-related factors [37].

The main sources of melatonin in the gut are some selective intestinal cells and intestinal bacteria, in addition to enzymes and diet [36]. Immunohistological studies found that enterochromaffin cells are the major producers of melatonin [38]. Also, the presence of enzymes responsible for melatonin biosynthesis was reported in the gut, such as serotonin-N-acetyltransferases or hydroxyindole-o-methyltransferases (HOIMT) [39]. Even though the pineal gland is the primary source of melatonin, its removal does not affect the melatonin levels in the intestines [27]. Nevertheless, these levels are known to be affected by age, as documented in mice, where higher levels of melatonin are reported in mice with older age [40]. In addition to its main function, melatonin in the gut serves as a transporter of some electrolytes such as sodium (Na^+^), potassium (K^+^), and calcium (Ca_2_^+^), due to its location in the intestinal villi [36]. Furthermore, advanced studies showed that melatonin helps in the contraction of smooth muscles of the stomach, ileum, and colon [41]. These findings confirm the multi-functional role of melatonin in the protection against gastrointestinal disorders.

Melatonin can act either on receptors or exert its function independently. The two major receptors are the Melatonin-1 receptor (MT1) (high affinity) and the Melatonin-2 receptor (MT2) (low affinity) [42]. The two are closely similar to each other and are derived from the G Protein-Coupled receptors (GPCRs) family. In addition, the melatonin-3 receptor (MT3) is another receptor implicated in the detoxification of xenobiotics, and it is known as quinone reductase 2 (QR2); it allows melatonin to exert a protective role against oxidative stress, a prominent factor in the etiology of IBD and CRC [43]. By acting on MT2, melatonin enhances cholecystokinin (CCK) production, which decreases intestinal motility by relaxing the gut muscles and attenuating the effect of serotonin [36]. Concerning absorption, melatonin helps decrease cholesterol absorption on the one hand and enhances the absorption of amino acids by stimulating the expression of their transporters on the other hand [44].

Table 1 illustrates the characteristics of each MT receptor mentioned above. Despite the important interaction of melatonin with these three colon receptors, it is worth mentioning that melatonin can interact with other receptors like nuclear receptors and cholecystokinin B receptor (CCK2) [45].

## 4. Melatonin and GM Interplay

### 4.1. Melatonin and Dysbiosis

In eubiosis, GM plays a significant role in protecting the GIT, maintaining normal homeostatic physiology, and, thus, sustaining the well-being of the gut [46]. The GIT is immediately colonized by bacteria after birth, and the microbiota undergoes dynamic changes during the first 1000 days of life, as it is influenced by dietary habits and environmental factors [47]. The alteration of the microbiota, termed dysbiosis, was directly associated with GIT infections and IBD [48]. In this respect, melatonin was shown to have a protective effect on GIT. It was demonstrated to regulate GM dysbiosis, which reduced GIT inflammation in experimental settings [49]. A recent study done on an experimental group of mice has shown that the appropriate maternal intake of melatonin can influence the gut microbiota of the offspring in mice and can protect them from early-life inflammatory diseases [50]. Moreover, the therapeutic effect of melatonin was also noticed due to its ability to modulate GM by enhancing eubiosis, which eventually improves the outcomes of dysbiosis-related diseases like IBD [37]. Additionally, the administration of a melatonin analogue has shown to have beneficiary effects on managing obesity in mice, mainly through the improvement of gut dysbiosis [51].

Moreover, GM dysbiosis was also associated with various diseases outside the GIT. For instance, patients with multiple sclerosis (MS) were found to have a higher incidence of dysbiosis when compared to controls [52]. This finding has led researchers to study the therapeutic effects of melatonin on improving MS outcomes due to its ability to improve dysbiosis [53]. A similar mechanism of interaction between melatonin and GM dysbiosis was studied in the pathogenesis of Alzheimer’s disease and obesity, further affirming the imperative role of melatonin in modulating dysbiosis, enhancing eubiosis, and preventing diseases both inside and outside the GIT [54].

### 4.2. Circadian Rhythm and Microbiota

In response to light-dark exposure in the eye, a neuronal circuit is stimulated to alter melatonin release from the pineal gland; its secretion is increased during darkness and decreased during light. In turn, melatonin regulates the body’s circadian rhythms, including sleep and hunger [55]. Nevertheless, it was found that gut melatonin levels were not directly affected by the pineal gland, and pineal melatonin, as mentioned before, was not the only source of secretion for this biomolecule. This was supported by studies using rats that underwent pinealectomy, where no significant alterations of gut melatonin levels were noted between rats with and without the pineal gland. However, only differences in serum melatonin concentrations were seen [56]. Further findings suggest that gut melatonin does not have a cyclic secretion. Rather, it is affected by the periodicity of food intake [57]. Studies have shown that the suprachiasmatic nucleus (SCN) of the hypothalamus is the center of integration of non-rhythmic circadian clocks throughout the body in order to synchronize them and provide a certain rhythmicity to different systems [58]. In turn, melatonin induces negative feedback on the SCN through MT1 and MT2 receptors, which contributes to the important role in phase-shifting [59,60].

On the other hand, it is well documented that food intake possesses modulatory effects on the gut microbiota. For instance, protein intake, digestion, and absorption affect the composition and function of microbiota [61]. Moreover, food intake was found to increase serum melatonin levels, which makes food a noteworthy regulator of circadian rhythm. This interference predisposes the GIT to several diseases mainly by altering the defensive effects of melatonin, and it emphasizes the importance of eating time in protecting from various GI pathologies [62,63].

Furthermore, the relation between gut microbiota and circadian rhythm alterations was more evident upon observing the GIT disturbances in sleep-deprived individuals. The selective overgrowth of gut bacteria was found to be a causative agent of sleep loss-related metabolic disturbances, and the use of probiotics actually improved the sleep quality of these persons [64]. In fact, studies on rats found that specific bacteria were linked to each chronotype [65]. Additionally, the structure, composition, and function of the gut microbiome were seen to be altered in mice with chronic jet lag, which also allowed the progression of fatty liver disease [66]. Hence, the gut microbiota is seen to have circadian oscillations, which confirms the need for further research to better understand this critical interplay [67].

### 4.3. Anti-Oxidation Effect of Melatonin

Melatonin has been shown to regulate oxidative stress balance within cells of different body systems. This effect gave it both protective and therapeutic properties [68], a phenomenon that was studied in various physiologic and pathologic states.

**Figure 2:**
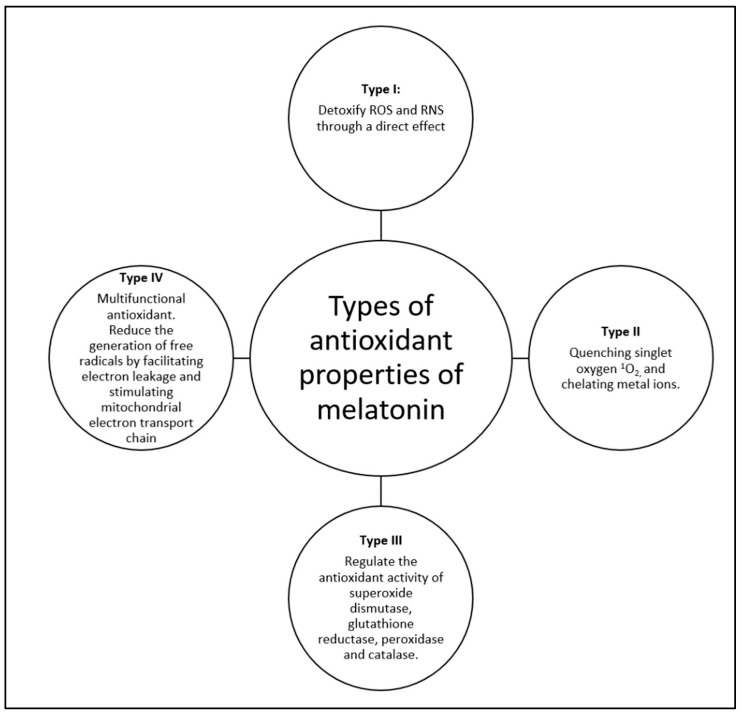
Summary of the role of melatonin as an antioxidant. ROS: reactive oxygen species, RNS: reactive nitrogen species. Adopted from Bonmati–Carrion et al. and Iesanu et al. [38,75].

For instance, it was shown that sleep deprivation, which is a cause of body melatonin-level dysregulation, was associated with altered intestinal mucosal barrier integrity and intestinal goblet cells (GC) dysfunction. Melatonin therapy has reduced the level of endoplasmic reticulum stress in GCs and improved the intestinal barrier function in sleep-deprived mice [69]. Additionally, treatment with melatonin was found to have beneficial effects in treating colitis in sleep-deprived mice through acting on the MT2 receptor, which leads to diminished levels of reactive oxygen species (ROS) [70]. Colitis in sleep-deprived mice was found to be caused, but not uniquely, by corticosterone. In such cases, it has been suggested that melatonin would be beneficial as a personalized targeted therapy [71]. In another study where rats underwent melatonin-treated fecal transplant, the degree of inflammation and autophagy was diminished by reduced oxidative stress when compared to controls, thus enhancing the role of melatonin in gut wellbeing and maintenance of GM [72]. Moreover, the effect of melatonin in regulating inflammation and oxidative stress has made it a promising intervention in controlling obesity [73]. Figure 2 summarizes the antioxidant role of melatonin in the gut. In addition to its multifunctional role as an antioxidant, melatonin undergoes enzymatic degradation and interacts with free radicals, which consequently generates hydroxylated melatonin metabolites that play a crucial role in antioxidation. Therefore, the antioxidative effect of melatonin and its metabolites makes the cascade of radical scavenging never-ending [74].

## 5. Melatonin and IBD

### 5.1. Intestinal Barrier, Melatonin, and IBD

Several studies documented the protective role of melatonin on intestinal barrier functions [76]. The destruction of the mucosal barrier, which leads to an increase in intestinal permeability, is one of the key events in IBD pathogenesis [76]. Various studies showed that the mucosal layer in active IBD is thinner and more discontinuous, the number of goblet cells is reduced, and the expression of some proteins, such as those involved in maintaining tight junctions, including adherens junction components (claudin 5 and 8, junctional adhesion molecule-A (JAM-A), and zonula occludens 1 (ZO-1)), are attenuated [77,78]. Sleep deprivation could lead to a decrease in melatonin and consequently to an impairment in tight junction proteins by changing melatonin levels, thus leading to leakage in the intestinal barrier and influx of the luminal contents, such as transepithelial invasion could induce an inflammatory reaction, which could be the initial factor in IBD pathogenesis [10]. Moreover, studies demonstrated that the reduction of adiponectin expression in colitis mice models is also triggered by sleep deprivation [79]. Melatonin administration increased this expression and, hence, ZO-1 and Occludin [78]. Such events improve the integrity of the intestinal barrier. Similarly, other studies showed that sleep deprivation for 3 days could lead to intestinal mucosal damage, yet treatment with melatonin ahead of the development of colitis significantly lowered inflammation and decreased colon damage [80]. In addition, melatonin lowered intestinal permeability by attenuating the effect of ethanol on receptors [81].

Multiple studies on rat models of colitis targeted colon injury, and various results concerning the effect of melatonin on IBD were reported. For example, treatment with melatonin reduces Tumor Necrosis Factor alpha (TNF-alpha) in serum and significantly decreases colonic injury [82]. In addition, melatonin protects epithelial cells and lowers para-cellular permeability by lowering the effect of Interleukin 1 Beta (IL-1 beta), increasing the anti-inflammatory effect in the gut and enhancing the role of IL-10. This has led to the strengthening of the epithelial barrier [83]. In addition, melatonin has a potent role as an antioxidant, which moderates molecular pathways in inflammatory responses in IBD [84] and has the potential to control oxidative stress and cell injury [85].

The critical role of the gut mucosal barrier in IBD pathogenesis, in addition to the direct effect of melatonin on intestinal mucosa, highlights the need for further studies to better understand this relationship.

### 5.2. Intestinal Microbiota, Melatonin, and IBD

**Figure 3:**
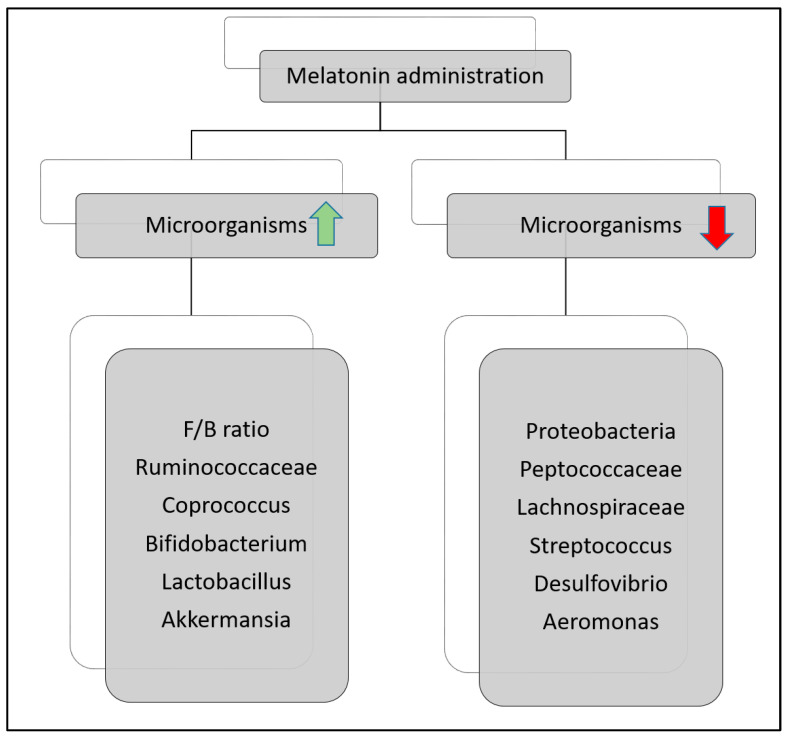
Alteration of the gut microorganisms after administration of melatonin. The green arrow indicates the increase in microorganisms, and the red arrow indicates the decrease in the microorganisms listed below. Adopted from Iesanu et al. [38].

The bacterial composition of the gut in patients with IBD undergoes many changes and modulations in quantity and quality, which creates an imbalance between inflammatory and anti-inflammatory pathways [86]. Replenishment of missing strains with probiotics could transform dysbiosis into eubiosis. For instance, Lactobacillus acidophilus is used as a treatment for IBD as it can decrease the production of pro-inflammatory cytokines induced by Th17 cells, such as IL-6, IL1-beta, IL-17, and, at the same time, increase the production of T-reg cells and IL-10 [87,88]. A close correlation was found between melatonin and intestinal bacteria, where injecting melatonin in mice increased Firmicutes/Bacteroidetes (F/B) ratio and reversed the lack of protective bacteria present as a result of IBD, thus regaining enbiosis [89]. Briefly, the administration of melatonin altered the gut microbiota composition by increasing microorganisms that act as probiotics and help in the treatment of IBD, and by decreasing some species favoring the pathophysiology of UC and Crohn’s disease, Figure 3.

Additionally, what supports the hypothesis of the link between IBD and melatonin is the increase in Lactobacillus and Akkermansia in the intestinal tissue of mice after treatment with melatonin, [89,90]. Nevertheless, melatonin is further involved in IBD-related dysbiosis; a recent study showed a significant decrease in the growth of *Escharichia coli* (*E.coli*) after treatment with melatonin [91]. This resulted in the attenuation of the inflammatory response generated by this bacterium and a decrease in serum TNF-alpha levels [91]. In brief, the relationship between melatonin and intestinal bacteria is complex. On one hand, the gut microbiome modulates the intestinal synthesis of melatonin and, on the other hand, melatonin alters the bacterial composition of the gut [37]. Table 2 shows the influence of gut bacteria on melatonin and how the latter is influenced by microbiome alteration.

Furthermore, melatonin and gut-derived metabolite interact together. Table 3 simplifies the interplay between some metabolites and melatonin [37].

Overall, melatonin remains an important factor in treating IBD by either increasing bacteria species responsible for attenuating IBD or by inhibiting the growth of destructive bacteria, a topic that necessitates further exploration.

## 6. Melatonin as Anticancer Medication in CRC

Many studies have proved melatonin to have anticarcinogenic properties through several mechanisms. It is worth mentioning that most of the mechanisms that are mentioned next are experimental, with little clinical application until today. Table 4 summarizes the outcomes of recent studies evaluating the effect of melatonin on CRC by acting on different pathways. The mentioned outcomes will be more developed in the upcoming sections.

### 6.1. Proliferation Inhibitors

The interaction between the cancerous cells and the tumor microenvironment has been implicated in tumor proliferation, invasion, and metastasis. Cancer-associated fibroblasts are crucial contributors to changing the microenvironment, mainly through growth factors and cytokines release [116]. By regulating the interactions between cells, cells and extracellular matrix on one hand, and enhancing the chemotherapeutic effect by decreasing chemoresistance on the other hand, melatonin has been proven to be effective in decreasing cancerous cellular proliferation [117].

Melatonin has also been shown to be beneficial as an adjunct therapy in treating colorectal cancer (CRC) through various pathways. For instance, tissue expression of Zinc Finger Protein 746 (ZFP746) was demonstrated to be increased in CRC when compared to noncancerous colorectal tissues, and it contributed to CRC cell proliferation. Upon targeting cancerous cells with 5-Fluorouracil (5-FU), melatonin use as an adjunct drug showed decreased cell resistance to 5-FU, mainly through suppression of this signaling pathway [118].

**Figure 4:**
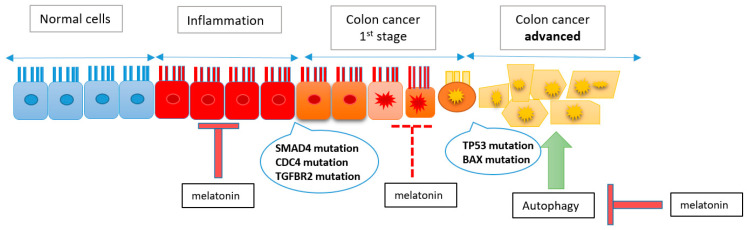
Effect of melatonin on colon cancer progression. Red arrows indicate the inhibitory effect of melatonin. The dashed arrow describes the possible effect of melatonin in inhibiting the progression of early stages of cancer to advanced ones. The green arrows report the stimulation of autophagy favoring the development of advanced colon cancer.

Melatonin was found to act on colon cancer cell proliferation by inhibiting the progression of inflammation to the early stages of cancer and by inhibiting autophagy [119]. However, a possible effect of melatonin has been reported in inhibiting the progression of early colon cancer to advanced stages [120]. Thus, further investigations are needed to explore how melatonin acts. Figure 4 illustrates briefly how melatonin acts on different steps in colon cancer progression.

Moreover, melatonin was also studied as a therapeutic agent for another gastrointestinal tumor, gastric cancer, and it exhibited this effect by acting on the exosomal miR-27b-3p-ADAMTS5 pathway [121]. It was also demonstrated to have the same effect by targeting another signaling pathway, the myosin light-chain kinase (MLCK) pathway [122].

### 6.2. Apoptosis Activation and Melatonin

Another way of opposing cancer progression by using melatonin is the activation of apoptosis in cancerous cells. Autophagy helps cells to maintain their homeostasis and integrity by getting rid of unwanted intracellular components, which usually result from cellular stress. When exaggerated, this mechanism can lead to cell death, termed apoptosis [123]. The fine balance in this pathway is implicated in the pathogenesis of various diseases, including cancers, and is a target for different therapeutic agents, including melatonin [124].

Melatonin was documented to be beneficial in treating CRC using this mechanism. For example, melatonin increased the sensitivity of CRC cells to 5-FU by enhancing apoptosis in these cells through the regulation of the miR-532-3p/β-catenin pathway [125]. It has also shown promising results of apoptosis induction in CRC when used synergistically with another natural product, Andrographis [126]. Additionally, cellular prion proteins (PrP^c^) were found to have a role in CRC growth and progression. In turn, melatonin acts on inhibiting the PrP^c^-dependent pathway, which increases superoxide production and induces apoptosis of cancerous cells [114,127]. The same pathway was also involved in oxaliplatin resistance in CRC cells, in which the therapeutic combination of melatonin and oxaliplatin was shown to have a significant effect in decreasing tumoral resistance [128]. Moreover, some studies suggested that melatonin acts by regulating MicroRNAs where it increased MicroRNAs expression, which eventually induced apoptosis in tumoral cells [119].

### 6.3. Angiogenesis Inhibition and Melatonin

The tumoral vascular supply provides the needed support for the growth and expansion of cancerous cells. Multiple treatment regimens have been explored to inhibit angiogenesis, and they are usually given in conjunction with other drugs to achieve better efficacy [129]. This oncostatic mechanism is attained using melatonin in several types of cancers [130]. In more detail, the tumoral cells mediate angiogenesis through secreting hypoxia-induced factor 1α (HIF-1α), which in turn acts by upregulating vascular endothelial growth factor (VEGF), the most important key player in angiogenesis. Melatonin exerts its antiangiogenic properties mainly by intervening with this pathway [131].

For instance, melatonin was found to decrease cell viability of metastatic CRC cells when used in combination with andrographolide, as mentioned before, and by also having antiangiogenic properties [126]. The combination of melatonin and andrographolide significantly decreased the microvascular extent and progression of the tumor [132]. Additionally, other experimental studies have shown that melatonin was able to decrease the CRC microvascular density and inhibit the growth and metastasis of CRC in the liver when used alone [133]. Moreover, it was shown that endothelin-1 (ET-1) plays a role in CRC growth and expansion when activated. In this context, melatonin was found to inhibit the ET-1 effect and, subsequently, offset angiogenesis and inducing apoptosis of tumoral cells [105].

### 6.4. Melatonin as an Antioxidant

In normal physiologic states, there is a cellular balance of pro-oxidant and antioxidant agents in order to maintain optimal beneficial oxidative stress levels. The higher basal levels of reactive oxygen species (ROS), the persistently high oxidative stress, and the imbalance between these oxidant agents are a key component of carcinogenesis in several tumors [134]. In turn, melatonin has also been implicated in this mechanism [68].

For instance, melatonin was proved to have a protective effect on normal colon cells in mice by regulating DNA damage and oxidative stress damage, which in turn prevented CRC development [135]. On the other hand, the combination of melatonin and menadione, a synthetically produced vitamin K analogue has shown to increase the superoxide content and the formation of nitrogen reactive species within the CRC cells [136]. Additionally, melatonin was found to act on the endoplasmic reticulum (ER) stress pathway by increasing reactive oxygen species, which eventually leads to the apoptosis of tumoral cells [137]. Furthermore, melatonin was studied in mice with CRC upon receiving radiation therapy. It proved to have a protective role in healthy tissues where it diminishes the damage caused by irradiation, and an enhancing effect in sensitizing tumoral cells to radiation, all by regulating the cellular oxidative balance [138].

## 7. Immune System, Melatonin, and IBD

### 7.1. Immunomodulation

Over the past decade, immunotherapy has been gaining great interest among researchers and clinicians, as it is shown to be a very promising therapeutic modality in treating several types of inflammatory diseases and cancer, including CRC [139]. Melatonin has been proven to possess important immunomodulatory properties, which also highlights the importance of adopting it as an antitumoral agent [140].

Apart from the pineal gland, melatonin is secreted by other cells, including different immune cells [120]. For instance, melatonin is implicated in T-cell differentiation and activation. It is needed especially for Th17, Treg cells, and memory T cells. This mechanism plays a role in both protecting the body against several types of cancer and in fighting the already established and metastatic cancers [141].

Previous sections highlighted the crucial role of the intestinal microbiota and how the intestinal bacterial flora disrupts the mucosal barrier, causing inflammatory reactions. These changes can lead to modulation and dysregulated immune system in IBD [142]. Research data stressed the role of the immune system in IBD and CRC, starting with the innate response to the acquired immunity mediated by cytokines, dendritic cells, and T cells (CD4+ effector and regulatory T cells) [143,144]. Cytokines have a key role in IBD via innate and adaptive immune cells by determining the differentiation of T cells either to Th1, Th2, or T regulatory cells [145]. Thus, proinflammatory cytokines lead to the destruction of tissue and the progression of IBD [146]. As discussed, IBD produces numerous pro-inflammatory cytokines (e.g.,: TNF-alpha) mediated by NF-kB; this signaling pathway plays a major role in the progression of IBD [146]. 

On the other hand, factors responsible for melatonin synthesis, such as the enzymes arylakylamine-N-acetyltransferase (AANAT) and tryptophan hydroxylase (TPH), are present in immune cells [147]. Those enzymes, and those receptors, make melatonin an “immune buffer” [147]. A study done by Vico et al. [148] suggested that melatonin could either act as a stimulant in patients suffering from immunosuppression or as an anti-inflammatory factor.

### 7.2. Neutrophils

Several studies demonstrate an important role of the innate immunity in IBD pathogenesis [149]. There are no conclusive results concerning the role of neutrophils in IBD, yet some studies found that colitis could be reduced with anti-neutrophil antibodies, while other studies showed that colitis is aggravated after depleting the number of neutrophils by using antiserum or by inhibiting their adhesions with anti-L-selectin mAB [150]. However, studies on humans showed that the increase in neutrophils is one of the histological parameters of IBD pathogenesis [151].

Furthermore, neutrophils are increased in sleep-restricted patients, resulting in an impairment of phagocytosis and dysregulation of the NADPH oxidase activity of the neutrophils [152]. In its turn, melatonin could influence neutrophils in many respects. For instance, research on rats concluded that melatonin attenuates the infiltration of neutrophils in the gut [82]. The function of L-selectin, molecules, which play a key role in leukocyte chemotaxis, is inhibited by the presence of melatonin [153]. Additionally, N-acetyl-N-formyl-5-Methoxykynuramine (AFMK), a melatonin metabolite present in rat brains, was found to have some antioxidative effects by decreasing the production of pro-inflammatory cytokines [154]. All these findings suggest a crucial role of melatonin on neutrophils and, perhaps, on IBD.

One of the recent theories suggests that the production of melatonin is increased in colon tissue during IBD flares [10]. To confirm, a study conducted on active cases of ulcerative colitis showed that hydroxyindole-O-methyltransferase (HIOMT), the enzyme that is responsible for the last step of melatonin biosynthesis was increased in the colon, indicating an enhancement of melatonin production in the gut during active IBD [148].

Moreover, several studies on rats demonstrated that daily treatment with 10 mg/kg intra-rectal melatonin could significantly ameliorate the Nancy score, which assesses the activity of colitis using histology [155], especially in samples taken from the ascending colon [156]. Altogether, these findings confirm the role of controlling the inflammatory reactions of melatonin in reducing IBD flares. However, further studies are needed to better understand its mechanism and action in this particular situation [157].

### 7.3. Macrophages

Side by side with neutrophils, the activity of macrophages is also influenced by melatonin [158]. Macrophages are divided into two main types; M1-Macrophages, possessing pro-inflammatory characteristics [159], and M2-Macrophages, possessing anti-inflammatory effects [160]. M1-Macrophages can produce a large amount of pro-inflammatory cytokines, such as *IL-1Beta*, *IL-23*, *IL-6*, and TNF-alpha, while M2-Macrophages secrete anti-inflammatory cytokines such as *IL-10* and TGF-Beta [161,162]. While M1-Marophages present the antigen and stimulate Th1 and Th17, M2-Macrophages regulate the immunity through stimulating Th2 response and mediating tissue repair [160,163]. The balance between M1 and M2 macrophages plays a major role in shaping the disease evolutions in IBD [160].

Experiments have shown that colitis models had a decrease in M2-macrophages and an increase in M1-Macrophages, with their respective cytokines [164]. The inhibition of M2-Macrophage polarization exacerbates colitis, making the shift from M1 to M2 a widely studied therapeutic approach for IBD [164].

Discontinuous sleeping and stressful situations were found to increase the number of M1 macrophages [165], suggesting a potential role for melatonin on this axis. This was confirmed by some studies where melatonin significantly decreased M1-Macrophages and attenuated their pro-inflammatory response [166]. This effect was established by the activation of STAT3 signaling responsible for the accumulation and activation of immunosuppressive cells (e.g: Treg, Th17) [167].

To sum up, such findings suggest melatonin to be an essential modulator in IBD by acting on the differentiation of macrophages into pro- or anti-inflammatory phenotypes. Therefore, therapeutic strategies could build up on this role of melatonin in controlling inflammation through the balance between Macrophages M1 and M2.

### 7.4. Natural Killers (NKs)

Another important involvement of melatonin in the innate immune response related to IBD progression is through the natural killers (NKs) [149], which are regulatory cells that can amplify inflammation [168]. Nevertheless, the role of these cells appears to be blurry in the pathophysiology of IBD [168]. Despite the low number of studies done, a study revealed that the frequency of CD16+, produced by the NK cells in the lamina propria, was higher in patients with IBD compared to healthy ones [169]. In light of this, Azathioprine and 6-mercaptopurine, common drugs used in the management of IBD [170], influence NK cells by decreasing their number, reducing INF-Gamma, and attenuating cytotoxicity of those cells [171,172]. Moreover, Killer Immunoglobulin-like receptor genes (KIR genes), which are receptors present on the surface of NK cells, play a role in IBD pathogenesis [173]. The link of melatonin to NKs is not clear yet. However, the presence of melatonin receptors (MT-1) and HIOMT in NKs could be a start for a better understanding of this relationship [174].

Till our day, there are numerous contradictions in the effect of melatonin on NK cells: an in vitro study showed that melatonin decreases the activity of NK cells, while an in vivo study showed a rise in the activity of those cells following treatment with melatonin in mice [175,176]. A significant increase in the number of these cells, especially in the spleen and bone marrow, has been proven by Coppin et al. [177]. Moreover, multiple studies suggest that sleep disorders increase the activity of NKs [178].

Further experiments are needed to better understand the relationship between NK and melatonin in IBD and to assess any possible role in CRC.

### 7.5. T Lymphocytes

In addition to the innate immunity, melatonin was found to affect the adaptive immune response by acting on CD4+ T cells [179]. These cells have melatonin-associated factors on their surface, such as the receptors MT1, RZR-Alpha, and HIOMT [45]. Experimental studies showed that melatonin increased the proliferation of T cells [179]. Furthermore, a study on patients with myasthenia gravis found that melatonin was able to negatively affect Th1 response, hence, reducing the IFN-gamma production and TNF-alpha and increasing anti-inflammatory interleukins such as IL-4 and IL-10 [53,180].

All the outcomes of related studies suggested an important role of melatonin in regulating the adaptive immune response by acting on Th1 and Th2. A comparison between changes in the adaptive immune system caused by melatonin, and those happening in IBD, is summarized in Table 5 [148,181].

Melatonin can increase the expression of FoxP3 on CD4+ Treg cells, where FoxP3 is a factor that has major regulatory effects on these cells. In some cases, FoxP3 can decrease this expression in some gastrointestinal diseases [182]. On this basis, melatonin is expected to have a therapeutic role in treating IBD, a topic worth more exploration.

## 8. Conclusions and Future Perspectives

The multifunctional activities of melatonin make it a potential addition to the therapeutic armamentarium of IBD and CRC. Melatonin strengthens the intestinal mucosal barrier and changes its bacterial composition, leading to eubiosis and, consequently, some anti-inflammatory properties through the regulation of immune responses and attenuating inflammation. It also has a defensive role in attenuating oxidative stress and reducing ulcers. In addition, evidence supports the fact that the clinical use of melatonin could extend the remission period and improve the effectiveness of conventional treatment regimens in IBD. Furthermore, growing evidence has also focused on a possible benefit of melatonin in CRC, where its anti-cancer roles were majorly focused on regulating the homeostasis of the tumor microenvironment.

Taking into consideration the above-mentioned advantages of the use of melatonin in the management of IBD and CRC, studies assessing clinical aspects are sparse and mainly performed on a small sample. Moreover, the safety and efficacy of melatonin in therapeutic strategies is not well studied, in addition to its drug interaction and gut mobility. Also, several mechanistic pathways need to be more extensively studied to find out the potential uses of melatonin. More precisely, the onco-protective effect of melatonin mainly inhibits cancer metastasis by stimulating several mechanisms that are yet to be fully understood. Further investigation is needed regarding the stimulation of E-cadherin, reduction of MMP-9, and attenuation of the activity of Fox0-1 and NF-kb [183]. In addition, the ability of melatonin to target signaling pathways in cellular proliferation and metabolism also needs to be more extensively explored, with its potential to inhibit the activity of COX-2 and iNOS, thus reducing the expression of proinflammatory genes. This can lead to a multimodal approach against the progression and metastatic spread of CRC [184]. Moreover, the role of melatonin in reversing the resistance and harmful effects caused by the combination of chemotherapy and radiotherapy in treating CRC is an important field to study [185]. Furthermore, the findings of some studies carried out on mice models regarding the inhibitory role of melatonin on the function of the tumor suppressor p53 gene highlights the need for further investigation in this direction [185].

Despite the rich literature that highlights the effect of melatonin on IBD and CRC, the transition from animal models into clinical aspects is not well studied, necessitating further studies focusing on applications in humans, with the aim of reaching a definitive adjuvant role for melatonin in the management of IBD and CRC.

## Figures and Tables

**Table 1:** Characteristics of melatonin receptors present in the gut [36,37].

Melatonin Receptor	MT1	MT2	MT3
Previously named	Mel1A	Mel1B	Mel1C
Coupled Receptors/Binding site	G protein-coupled Receptors		Quinone reductase 2 (QR2)
Function/mechanism	− Mediates adenylate cyclase inhibition and phospholipase c beta activation	− Phosphoinositol production − Inhibition of adenylate cyclase − Inhibition of soluble guanylate cyclase pathways	− Blocked by prazosin − MT3/QR2 activation protects against oxidative stress
Abundance	Ileum, colon and mucosa	− Muscularis mucosa and submucosa of Ileum and colon − Muscularis propria of colon	Ileum and Colon

**Table 2:** Effect of some species present in the gut on melatonin.

Species	Influence of Gut Bacteria on Melatonin and Melatonin Precursors	References
*Helicobacter pylori* (*H.Pylori*)	− Downregulation of AANAT and ASMT − Reduction in melatonin production	[38]
*Lactobacillus rhamnosus*	− More melatonin receptor genes in Zebrafish	[92]
*Clostredium sporogenes* *Ruminococcus gnavus*	− Convert Trp to Tryptamine (metabolite)	[93]
*E.coli* *Bacteroides*	− Metabolization of Trp	[94]
*E.coli* or *Lactobacillus rhamnosus*	− Decrease melatonin levels in serum − Increase melatonin levels in colon to alleviate lipidic dysmetabolism	[95]
*Roseburia hominis*	− Increase melatonin levels via its metabolites (propionate and butyrates)	[96]

**Table 3:** Interplay between melatonin and gut-derived metabolites.

Metabolites	Effect	References
SCFA	− Stimulate enterochromaffin cells, hence increasing melatonin in gut	[37]
TMAO	− Melatonin reverses the change of TMAO–TMA ratio induced in CKD.	[97]
*Aeromonas Veronii* LPS	− Melatonin inhibits neuroinflammation and ameliorates memory impairment	[98]
Bile acids	− Melatonin regulates bile acid metabolism	[99]

Legend: SCFA: Short chain fatty acid; TMAO: Trimethylamine N-oxide; TMA: Thrombotic microangiopathy; CKD: Chronic kidney disease; LPS: Lipopolysaccharide.

**Table 4:** Summary of outcomes of the recent studies demonstrating the effect of melatonin administration at different levels in the progression of Colon Cancer (CC).

Study	Effect of Melatonin
Gao et al., [100]	Contributes to the activity of 5-FU in inhibiting CC cell migration
Liu et al., [101]	Inhibits RKO CC migration by attenuating ROCK expression (through p38/MAPK)
Zou et al., [102]	Reduces CC cell proliferation by inactivating p38/MAPK
Anisimov et al., [103]	Reduces the depth of invasion of CC in vivo
Park et al., [104]	Reduces the expression of VEGF by destabilizing HIF-1*α* and by acting on HIF-1α activity in CC cells.
Léon et al., [105]	Inhibits angiogenesis by blocking ET-1 release from CC cells
Palidarova et al., [106] and Srinivasan et al., [107]	Affects the immunity by targeting specific cells (such as TH cell) to produce Interleukins to attenuate CC development
Kossoy et al., [108]	Acts on the lymphatic system of the host to exert its anti-carcinogenic effect
Farriol et al., [109]	Antiproliferative activity by decreasing cell growth in non-hormonal depedent colon cells
Winczyk et al., [110]	Oncostatic effect through MT2 receptors acting on RZR/ROR α nuclear receptors.
Hong et al., [111]	Induces colon cell death programs and stops mitotic activity through G1-phase arrest
Wei et al., [112]	Contributes to apoptosis in CC through HDAC4 nuclear import
Chovancova et al., [113]	Induces apoptosis in CC cells through Na/Ca exchanger type 1 and IP3 type 1 receptors/
Yun et al., [114]	Induces mitochondria-mediated cell apoptosis in CC cells through PrP^C^ pathway
Kannen et al., [115]	Limits CC progression by controlling malignant lesions in CC through CD68+ and CD133+ cluster cells

CC: colon cance; 5-FU: 5-fluorouracil; ROCK: Rho-associated coiled-coil kinases; VEGF: vascular endothelial growth factor; MAPK: mitogen-activated protein kinases; HIF: hypoxia inducible factor; ET-1: endothelin-1; HDAC: histone deacetylase.

**Table 5:** Effect of treatment with melatonin on different factors implicated in the adaptive immunity in patients with IBD.

Immune Factors/Cells	Effect of IBD	Effect of Melatonin
Th17	-	Inhibition of differentiation
T-reg	-	Increase production
IL-17	Increase in number	Remarkable reduction in number
IL-23	Increase in number	Mild reduction in number
IL-10	Reduction in number	Increase production
IL-6	Increase in number	Significant reduction in number
TNF-alpha	Increase in number	Significant decrease in number
CD4+T	Increased in number, main drivers of inflammation	Induces in-vitro proliferation

## Data Availability

Data are available upon request from corresponding author.
